# Peroxisomal activity drives aggressive bladder cancer phenotypes and reveals erythorbic acid as a potential therapeutic modulator

**DOI:** 10.3389/fonc.2026.1734437

**Published:** 2026-03-04

**Authors:** Qinghui Wu, Yu Zhou, Zhewen Ou, Housheng Fu, Fanchang Zeng, Daoyuan Li, Zhaocong Zheng, Fei Wang

**Affiliations:** 1 Department of Urology, Hainan General Hospital, Hainan Affiliated Hospital of Hainan Medical University, Hainan Provincial Clinical Medical Center, Haikou, Hainan, China; 2 Admission Service Center, Hainan General Hospital, Hainan Affiliated Hospital of Hainan Medical University, Hainan Provincial Clinical Medical Center, Haikou, Hainan, China; 3 Department of Radiation Oncology, Guangzhou Institute of Cancer Research, The Affiliated Cancer Hospital, Guangzhou Medical University, Guangzhou, China

**Keywords:** bladder cancer (BC), cancer metabolism, peroxisomes, stromal cells, tumor microenvironment (TME)

## Abstract

**Background:**

Peroxisomes play essential roles in cellular lipid metabolism and redox regulation, yet their contribution to bladder cancer (BLCA) progression remains poorly defined.

**Methods:**

Transcriptomic and clinical data from TCGA-BLCA and three GEO cohorts were integrated to identify prognostic peroxisome-related genes (PRGs). A six-gene PRG signature was constructed and validated for survival prediction, molecular subtype stratification, and pathway enrichment analyses, with expression validation in bladder cancer cell lines. Drug–gene enrichment and molecular docking were then performed to identify potential therapeutic modulators, which were subsequently assessed using CCK-8 cell viability assays.

**Results:**

Two distinct PRG-based molecular subtypes of BLCA were identified, showing significant differences in survival, mutational landscape, immune infiltration, and metabolic signaling. The high-risk subtype was enriched for PRDX1, ACOX2, and IDI1, reflecting enhanced oxidative stress adaptation and metabolic reprogramming, while the low-risk group was defined by ACSL5 and XDH. Drug-gene enrichment identified erythorbic acid, a redox-active ascorbate analog, as the most biologically relevant compound targeting high-risk PRGs. Molecular docking confirmed stable binding of erythorbic acid to ACOX2 (–6.2 kcal/mol), IDI1 (–6.6 kcal/mol), and PRDX1 (–5.4 kcal/mol) within catalytically active pockets, suggesting coordinated modulation of oxidative metabolism and redox balance. Subsequent CCK-8 assays demonstrated a dose- and time-dependent reduction in viability in bladder cancer cell lines. In contrast, normal urothelial XV-HUC-1 cells showed relatively preserved viability, indicating differential cellular responses to erythorbic acid *in vitro*.

**Conclusion:**

Peroxisome-related gene dysregulation shapes the metabolic and immunologic heterogeneity of bladder cancer. Erythorbic acid emerges as a promising redox-metabolic modulator targeting multiple peroxisomal enzymes, offering a potential therapeutic avenue for aggressive, high-risk BLCA subtypes.

## Introduction

According to the latest GLOBOCAN 2022 estimates, bladder cancer (BLCA) is the ninth most commonly diagnosed cancer worldwide, with approximately 614,298 new cases annually. It ranks as the 13th leading cause of cancer-related deaths and affects around 5.6 million people living within five years of diagnosis globally (1). BLCA is clinically classified based on tumor invasion into non-muscle-invasive bladder cancer (NMIBC) and muscle-invasive bladder cancer (MIBC). At diagnosis, approximately 75% of bladder cancer cases are NMIBC, which is a clinically heterogeneous disease characterized by high recurrence and, in high-risk cases, high progression rates (2). MIBC is marked by high recurrence, metastasis, and poor prognosis, with a 5-year survival rate of 60–70% (3). Despite significant advances over the past two decades—including laparoscopic and robotic surgeries, enhanced cystoscopy techniques, non-invasive biomarker urine tests, intravesical and systemic therapies such as immunotherapy, targeted therapy, and vaccine therapy, as well as novel surgical and drug delivery approaches—the 5-year survival rate for MIBC patients remains suboptimal (4). Therefore, there is an urgent need to identify novel biomarkers that can accurately predict prognosis and therapeutic response, thereby enabling personalized treatment strategies in BLCA.

Cancer cells undergo profound metabolic reprogramming to support their rapid growth, survival under stress, and evasion of immune responses (5). This reprogramming involves enhanced uptake and utilization of nutrients such as glucose, amino acids, and lipids to fuel biosynthesis, redox balance, and energy production (6). In bladder cancer, these metabolic shifts are well-documented (7). Tumor cells exhibit elevated glycolysis (the Warburg effect), increased lactate production, and reliance on glutamine metabolism for anaplerosis and redox control (8, 9). Mitochondrial adaptations, such as altered tricarboxylic acid (TCA) cycle activity and elevated reactive oxygen species (ROS) generation, further support the tumor’s metabolic plasticity (9, 10). These changes contribute to disease progression, chemoresistance, and poor prognosis.

Peroxisomes are dynamic, single-membrane-bound organelles that play essential roles in cellular metabolism (11). They are especially important for the β-oxidation of very-long-chain and branched-chain fatty acids, the synthesis of ether phospholipids, and the detoxification of hydrogen peroxide via catalase (11). In addition, peroxisomes regulate redox homeostasis and interact metabolically with mitochondria and the endoplasmic reticulum, influencing overall cellular energy balance (12). Emerging evidence highlights that peroxisomal functions are altered in many cancers, including bladder cancer (13). Increased expression of peroxisomal enzymes and transporters has been reported, suggesting an upregulation of lipid metabolic pathways to support membrane synthesis and signaling processes critical for tumor growth (11). Furthermore, the production and detoxification of ROS by peroxisomes may modulate signaling cascades involved in proliferation and survival (12). Given their central role in cellular lipid metabolism and redox regulation, peroxisomes are likely contributors to the metabolic reprogramming seen in bladder cancer. Understanding how peroxisomal pathways are co-opted in malignancy is crucial for identifying new metabolic biomarkers and therapeutic targets, particularly in cancers with high metabolic flexibility like bladder cancer.

This study aims to identify key peroxisome-related genes (PRGs) as diagnostic and prognostic biomarkers in BLCA and to classify patients into molecular subgroups based on PRG expression profiles using Cox regression, and random forest algorithms. By integrating transcriptomic data from The Cancer Genome Atlas (TCGA) and Gene Expression Omnibus (GEO) with pathway enrichment, mutational landscape analysis, tumor microenvironment profiling, and molecular docking, this research uncovers the functional role of peroxisomal activity in BLCA progression. The findings provide novel insights into BLCA metabolic heterogeneity, highlight erythorbic acid as a potential therapeutic drug, and support the development of more personalized prognostic and treatment strategies.

## Materials and methods

### TCGA data acquisition and preprocessing

Gene expression data for patients from the TCGA-BLCA project, including 41 normal and 487 tumor samples, were obtained using the *TCGAbiolinks* R package. A query was then constructed to retrieve Gene Expression Quantification data processed with the STAR - Counts workflow. FPKM-normalized expression values were obtained from the fpkm_unstranded assay provided within the GDC-generated SummarizedExperiment (**Supplementary Data S1**). Ensembl gene identifiers were converted to official gene symbols using a custom Perl script and a reference human GTF annotation file from the GENCODE project (https://www.gencodegenes.org/human/) (**Supplementary Data S2**). Duplicate gene symbols were resolved by retaining only the entry with the highest overall expression across all samples, resulting in a non-redundant, gene-level expression matrix suitable for downstream analyses.

### GEO data normalization and integration

For external validation, three independent bladder cancer cohorts were obtained from the GEO database: GSE13507 (GPL6102, n = 166), GSE32894 (GPL6947, n = 224), and GSE48276 (GPL14951, n = 73). Probe IDs from each platform were mapped to gene symbols using corresponding annotation files. Each dataset was independently processed using the limma package, with log_2_ transformation applied where appropriate and quantile normalization was performed. Genes common across all datasets were identified, and batch effects were corrected using the ComBat() function from the sva package. The resulting normalized and integrated expression matrix was used for downstream validation analyses.

### Peroxisome-related genes

To establish a comprehensive set of PRGs, we integrated data from three well-recognized and previously validated sources: PeroxisomeDB 2.0 (comprising 100 genes), the Kyoto Encyclopedia of Genes and Genomes (KEGG) pathway database (83 genes), and a proteomic dataset specific to human liver peroxisomes (60 genes) (14–16). After consolidating these datasets and eliminating redundant entries, we curated a final list of 113 non-overlapping PRGs, as detailed in **Supplementary Table S1**.

### Data integration, batch correction, and prognostic modeling

To enable integrative analysis, genes common to both TCGA and GEO cohorts were identified. Expression matrices were merged, and batch effects between TCGA and GEO samples were corrected using the ComBat function from the sva package (**Supplementary Data S3**). Shared PRGs were extracted from the corrected matrices and saved for downstream prognostic modeling. To develop the PRG-based prognostic signature, univariate Cox regression was used to identify survival-associated genes, followed by multivariate Cox regression to select independent prognostic markers. Risk scores were calculated as the weighted sum of gene expression and regression coefficients. Patients were stratified into high- and low-risk groups based on the median risk score.

### Prognostic biomarker identification and diagnostic gene selection

To identify prognostic biomarkers, univariate Cox proportional hazards regression was first performed to screen survival-associated genes (p < 0.05). Candidate genes were then further evaluated using a random survival forest model implemented with the randomForestSRC and randomSurvivalForest packages to assess prognostic importance. Genes with a relative importance score greater than 0.5 were considered prognostically relevant and retained for subsequent analyses. For diagnostic gene selection, a random forest classifier was applied, and feature importance was ranked using the Mean Decrease in Gini index, with top-ranking genes selected for downstream diagnostic evaluation.

### Somatic mutation profiling and tumor mutation burden estimation

Somatic mutation data for bladder cancer (TCGA-BLCA) were downloaded and processed using the TCGAbiolinks package. Mutation Annotation Format (MAF) files were curated and integrated with clinical risk information to stratify samples into high- and low-risk groups. Mutational profiles were analyzed and compared between groups using the maftools package. Mutations were further filtered to highlight selected signaling or metabolic pathways. Gene-specific mutation patterns for TP53 were visualized using lollipopPlot(), and co-occurrence or mutual exclusivity among frequently mutated genes was assessed using somaticInteractions() and coOncoplot(). Tumor mutation burden (TMB) was calculated using the tmb() function.

### Enrichment pathways analysis

To assess the enrichment of hallmark cancer pathways (KEGG Hallmarks) and immune-related pathways (curated gene lists; **Supplementary Table S2**) across PRG subtypes, single-sample Gene Set Enrichment Analysis (ssGSEA) was performed using the GSVA package in R. Normalized gene expression data, including log2 transformation and batch correction where applicable, were used as input to compute ssGSEA enrichment scores with the gsva function (method = “ssgsea”, kcdf = “Gaussian”, abs.ranking = TRUE) (**Supplementary Data S4**). The resulting ssGSEA scores for each gene set were scaled to the 0–1 range using min–max normalization performed per gene set. Differences in ssGSEA scores between groups were evaluated using the Wilcoxon rank-sum test, and the normalized ssGSEA matrix was used for downstream statistical analysis and visualization. To evaluate Gene Ontology (GO) and KEGG pathway enrichment between PRG subtypes, we utilized the clusterProfiler package in R, along with org.Hs.eg.db, by converting differentially expressed genes into Entrez IDs and applying enrichKEGG with p- and q-value thresholds set at 0.05. (**Supplementary Data S5**).

### Tumor microenvironment analysis

To comprehensively characterize the tumor microenvironment (TME), we first utilized the ESTIMATE algorithm via the “ESTIMATE” R package to infer tumor purity and quantify stromal and immune cell infiltration based on gene expression data (17). To estimate the absolute abundance of tumor microenvironment cell populations, we applied the MCP-counter method, which quantifies ten major cell types, including eight immune and two stromal populations, using predefined transcriptomic marker signatures. These signatures correspond to a fixed set of 112 curated marker genes implemented as the default gene sets in the MCP-counter R package (**Supplementary Data S6**; **Supplementary Table S3**) (18). These complementary approaches enabled a robust and multidimensional assessment of the TME landscape.

### Drug-gene interaction enrichment analysis using DSigDB

Drug–gene interaction enrichment analysis was performed using the Drug Signature Database (DSigDB) (https://dsigdb.tanlab.org/DSigDBv1.0/). Drug–gene association data were analyzed using the clusterProfiler package with gene annotation from org.Hs.eg.db to identify compounds significantly enriched for high-risk PRGs. Statistically significant candidates were selected based on p < 0.05 and FDR < 0.05 thresholds. The full list of enriched compounds was retained for transparency, while the final candidate was selected based on (i) biological plausibility, (ii) drug-likeness and safety, and (iii) structural tractability for docking analyses.

### Molecular docking analysis

To evaluate potential interactions between the lead compound and high-risk PRGs, molecular docking simulations were performed against three target proteins: ACOX2, IDI1, and PRDX1. The 3D structure of erythorbic acid was retrieved from the PubChem database (CID: 54675810) in SDF format. The 3D crystal structures of IDI1 (PDB ID: 2ICK) and PRDX1 (PDB ID: 7WET) were downloaded from the RCSB Protein Data Bank (PDB), while the structure of ACOX2 was obtained from the AlphaFold Protein Structure Database (AF-Q99424-F1-model_v6.pdb) due to the absence of an experimentally resolved model. All receptor structures were pre-processed by removing water molecules, co-crystallized ligands, and heteroatoms. Docking simulations were conducted using CB-Dock2 (https://cadd.labshare.cn/cb-dock2/index.php), which automatically detects binding cavities and ranks docking poses based on predicted binding affinity (Vina score). The top-ranked pocket for each protein was selected according to the lowest binding energy and the presence of catalytically relevant residues.

### Cell lines and cell culture

This study employed the human bladder cancer cell line T24 and the normal bladder epithelial cell line XVHUC1, both sourced from the Committee of Type Culture Collection, Chinese Academy of Sciences (Shanghai, China). Cells were cultured in Dulbecco’s Modified Eagle Medium (DMEM) supplemented with 10% fetal bovine serum (FBS), 100 U/ml penicillin, and 100 μg/ml streptomycin. All cultures were maintained in a humidified incubator at 37 °C with 5% CO_2_. Cell line authenticity was routinely confirmed through morphological assessment and regular mycoplasma testing to ensure contamination-free conditions.

### Quantitative real-time PCR

Total RNA was extracted and purified using TRIzol Reagent (Takara, Otsu, Japan), followed by reverse transcription to generate cDNA. Quantitative real-time PCR (qRT-PCR) was carried out using the SYBR Green PCR Kit (Takara, Otsu, Japan). GAPDH was used as an internal reference to normalize mRNA expression levels, and relative expression in the treated samples was compared to that of the control group. Primers were designed using Primer Premier 5.0 and Beacon Designer 7.8 software and synthesized by General Biosystems Co., Ltd. The primer sequences are provided in **Table 1**.

**Table 1:** Primers used in this study.

PRGs	Primers
ACOX2-F	AGCACCCCGACATAGAGAGC
ACOX2-R	CTGCGGAGTGCAGTGTTCT
ACSL5-F	GCTTATGAGCCCACTCCTGATG
ACSL5-R	GGAAGAATCCAACTCTGGCTCC
DHRS4-F	GAACTGCCTAGCACCTGGACTT
DHRS4-R	ACACGATGCCAGCACAATCCTC
IDI1-F	GCCGCAGACTGTGCTCAAAGC
DI1-R	CCTGTTGCTTGTCGAGGTGGTT
PRDX1-F	CTGCCAAGTGATTGGTGCTTCTG
PRDX1-R	AATGGTGCGCTTCGGGTCTGAT
XDH-F	GGACAGTTGTGGCTCTTGAGGT
XDH-R	GGAAGGTTGGTTTTGCACAGCC

### *In vitro* evaluation of erythorbic acid–induced growth inhibition in bladder cancer cells

Cell viability was evaluated using the Cell Counting Kit-8 (CCK-8) assay in the human bladder cancer cell lines T24 and HT-1197, as well as the normal urothelial cell line XVHUC-1. Cells were harvested by washing with phosphate-buffered saline (PBS) followed by brief trypsinization (1–2 min). After neutralization with complete culture medium, cells were gently resuspended to obtain a single-cell suspension and counted using a hemocytometer. The cell density was adjusted to 5 × 10^4^ cells/mL, and 100 μL of the suspension was seeded into each well of a 96-well plate. After 24 h of incubation, the medium was replaced with 100 μL of fresh medium containing the drug at indicated concentrations (0, 0.4, 0.8, 1.2, and 1.6 mM). Each concentration was tested in triplicate wells. Cells were then incubated under standard culture conditions, and cell viability was assessed by measuring optical density (OD) values at 24 h and 48 h following drug treatment according to the manufacturer’s instructions. Absorbance values were normalized to untreated control wells to calculate percent cell viability.

### Statistical analysis

Categorical variables were compared using the chi-square test, and continuous variables using the Student’s *t*-test or Wilcoxon rank-sum test, as appropriate. Kaplan-Meier survival curves were evaluated with the log-rank test, and correlations were assessed using Pearson or Spearman methods. A *p*-value < 0.05 was considered statistically significant. All statistical analyses were performed using R (v4.0.3).

## Results

### Overview of the PRG-based analytical workflow and key findings

**Figure 1:**
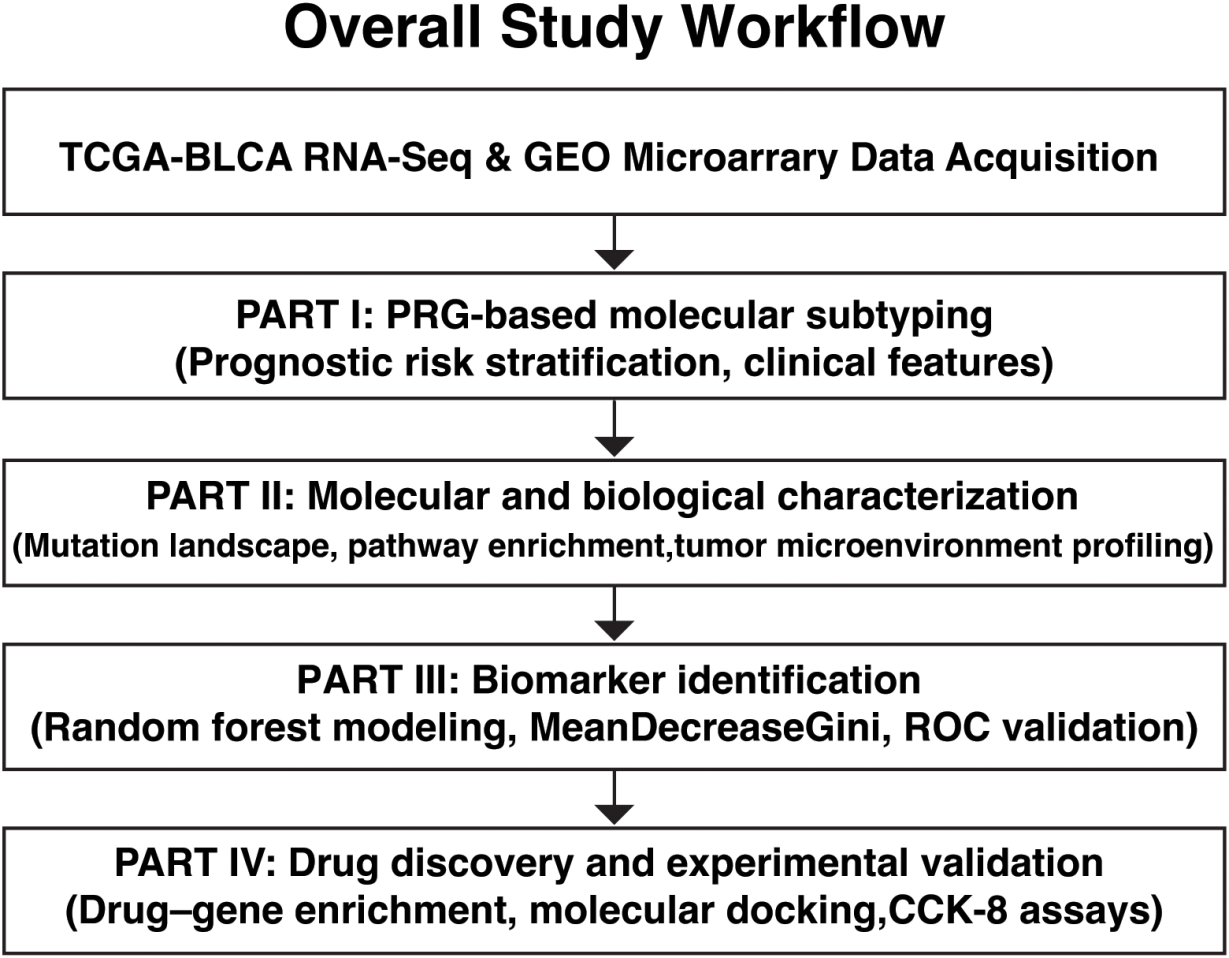
Schematic overview of the study pipeline. The workflow illustrates the four major analytical components of this study. (1) PRG-based risk subtyping: Transcriptomic data from TCGA-BLCA were used to identify prognostic PRGs through Cox regression analyses, resulting in a six-gene PRG signature that stratified patients into high- and low-risk molecular subtypes, which were independently validated in GEO cohorts. (2) Biological and clinical characterization: The two PRG-defined subtypes were comprehensively characterized by clinical features, somatic mutation landscapes, pathway enrichment analyses, and tumor microenvironment profiling using ESTIMATE and MCP-counter. (3) Biomarker identification: Machine learning and survival analyses were applied to identify key PRGs driving subtype differentiation and prognosis, highlighting PRDX1 and ACSL5 as representative biomarkers. (4) Therapeutic exploration and validation: Drug–gene enrichment analysis identified erythorbic acid as a candidate compound targeting high-risk PRGs, followed by molecular docking to assess binding affinity and *in vitro* CCK-8 assays to evaluate its inhibitory effects on bladder cancer cell proliferation.

The overall analytical workflow and key findings of this study are summarized in **Figure 1**. Using peroxisome-related genes (PRGs), bladder cancer patients were stratified into distinct molecular subtypes with different prognostic outcomes. These subtypes displayed marked differences in clinical features, mutation patterns, pathway activities, and tumor microenvironment characteristics, leading to the identification of representative PRG biomarkers and a candidate therapeutic compound with experimental validation.

### Identification of prognostic molecular subtypes in bladder cancer using peroxisome-related genes

**Figure 2:**
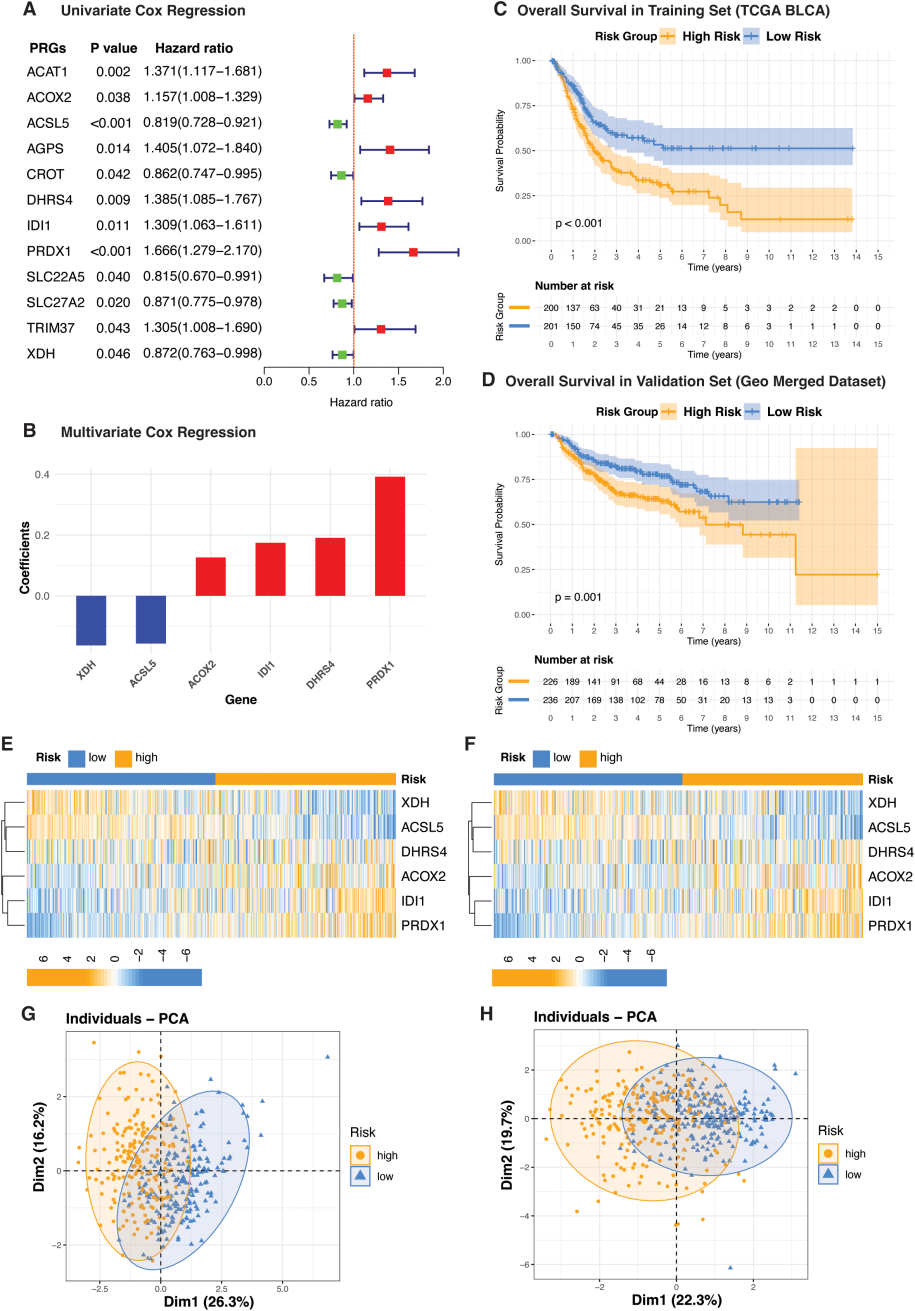
Risk stratification of bladder cancer based on peroxisome-related gene signature. **(A)** Forest plot showing the hazard ratios of peroxisome-related genes (PRGs) identified through univariate Cox regression analysis in the TCGA-BLCA cohort (n = 401). **(B)** Bar plot of regression coefficients from multivariate Cox analysis highlighting selected prognostic PRGs. **(C, D)** Kaplan–Meier survival curves comparing overall survival between high-risk and low-risk groups in the TCGA-BLCA cohort **(C)** and GEO validation dataset **(D)**. **(E, F)** Heatmaps of PRG expression patterns in the TCGA-BLCA cohort **(E)** and GEO validation cohort **(F)**. **(G, H)** Principal component analysis (PCA) illustrating the separation of risk groups based on the expression of six PRGs in the TCGA cohort (**(G)**, PC1: 26.3%) and GEO validation cohort (**(H)**, PC1: 22.3%).

To explore the prognostic role of PRGs in bladder cancer, we applied a two-step feature selection strategy involving univariate Cox regression followed by multivariate Cox regression. Transcriptomic data from the TCGA-BLCA cohort served as the training dataset (n=401), while a merged expression matrix compiled from three GEO bladder cancer cohorts (n=463) was used for independent validation. After integrating the datasets and retaining only overlapping genes, a total of 91 shared PRGs were identified for analysis. Univariate Cox regression analysis on the TCGA-BLCA cohort revealed 12 PRGs significantly associated with patient prognosis (**Figure 2A**). These were subsequently refined to 6 core prognostic genes using multivariate Cox regression (**Figure 2B**). Among them, four genes including Peroxiredoxin 1 (PRDX1), Isopentenyl-Diphosphate Delta Isomerase 1 (IDI1), Acyl-CoA Oxidase 2 (ACOX2), and Dehydrogenase/Reductase 4 (DHRS4) were classified as high-risk genes, as their elevated expression was associated with poor prognosis. In contrast, Acyl-CoA Synthetase Long Chain Family Member 5 (ACSL5) and Xanthine Dehydrogenase (XDH) were identified as protective, with higher expression linked to better survival in bladder cancer. Based on the expression levels and corresponding regression coefficients of these six genes, a risk score was calculated for each patient. Using the median risk score as a cutoff, patients in both the training and validation cohorts were stratified into high-risk and low-risk subgroups. This stratification revealed significant prognostic differences between the two subgroups in each dataset, supporting the ability of PRG-derived risk scores to define novel molecular subtypes of bladder cancer (**Figures 2C, D**). The expression profiles of the six PRGs across both datasets are shown in **Figures 2E, F**. Principal component analysis (PCA) confirmed the subgroup distinction, with the first principal component (PC1) accounting for 26.3% of the variance in the TCGA BLCA cohort and 22.3% in the validation dataset (**Figures 2G, H**).

### Clinical characteristics of BLCA PRG subtypes

**Figure 3:**
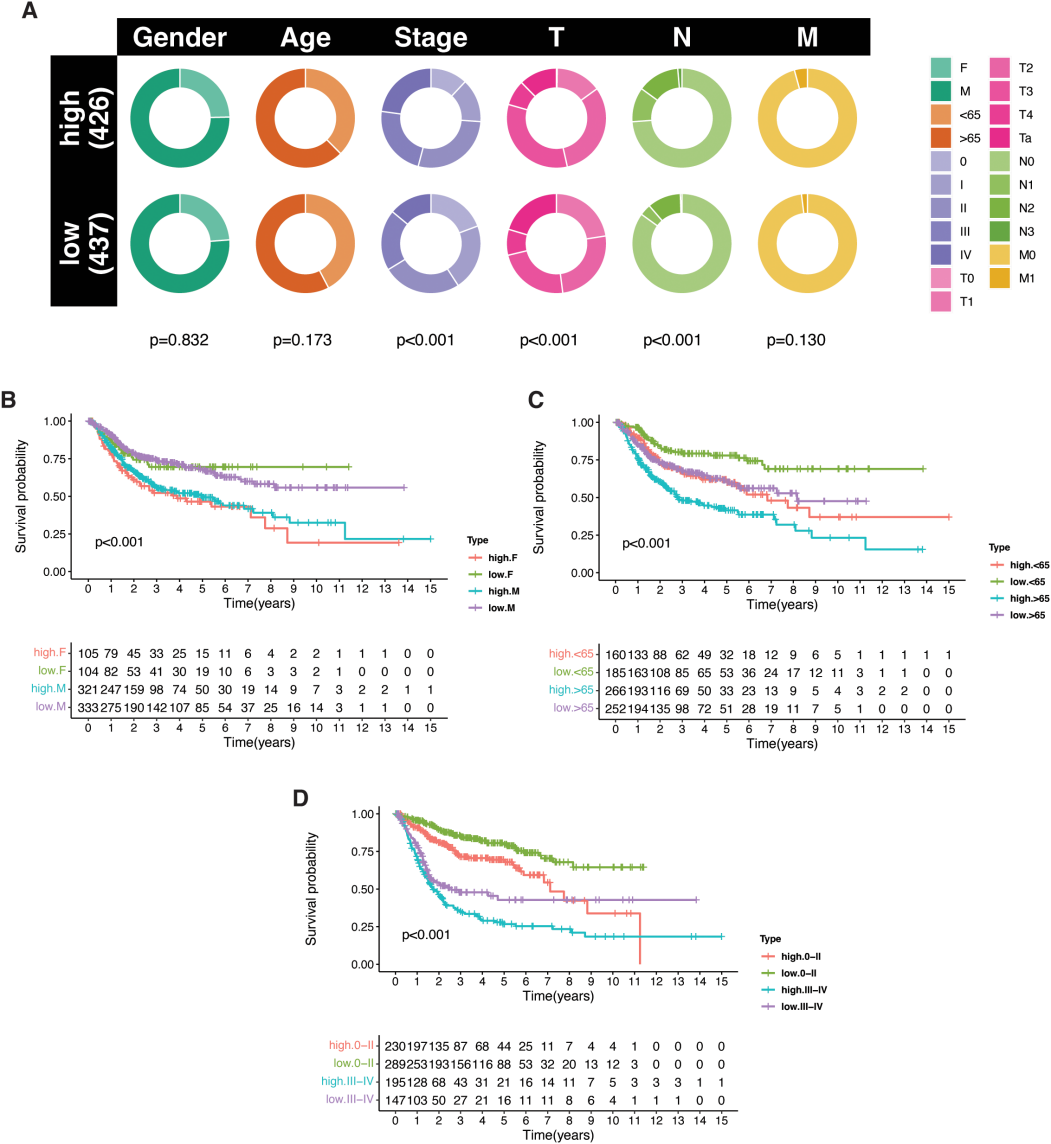
Clinical correlation and prognostic relevance of PRG-based subtypes in bladder cancer. **(A)** Circular plot illustrating the distribution of clinical features—age, gender, and tumor stage—across the identified PRG subtypes; statistical significance was evaluated using the chi-square test (P < 0.05). **(B–D)** Kaplan–Meier survival analyses demonstrating the prognostic value of PRG subtypes in combination with key clinical variables: **(B)** age (>65 *vs*. <65), **(C)** gender (male *vs*. female), and **(D)** tumor stage (stage 0–II *vs*. stage III–IV).

We next assessed the clinical characteristics associated with the identified PRG subtypes. No significant differences in age or gender distribution were observed among the PRG-defined subtypes (**Figure 3A**). However, marked differences emerged with respect to tumor stage, primary tumor size and extent, and lymph node involvement. The high-risk subgroup showed a greater proportion of patients with advanced-stage disease (stage III/IV) and extensive nodal metastasis. Additionally, tumors classified as T1-T3 were more commonly observed in the high-risk group, while early-stage tumors (Ta, stage 0) were more prevalent in the low-risk subgroup. Similarly, patients with one to three positive lymph nodes were more frequent in the high-risk category (**Figure 3A**). Notably, the PRG-based risk model retained its prognostic value across different clinical strata, including age, gender, and stage groups (0-II/III-IV), demonstrating its potential as an independent and robust classifier (**Figures 3B–D**). In conclusion, PRG subtyping effectively stratifies bladder cancer patients by tumor aggressiveness and provides meaningful prognostic insights beyond conventional clinical parameters.

### Mutation landscape in bladder cancer PRG subtypes

**Figure 4:**
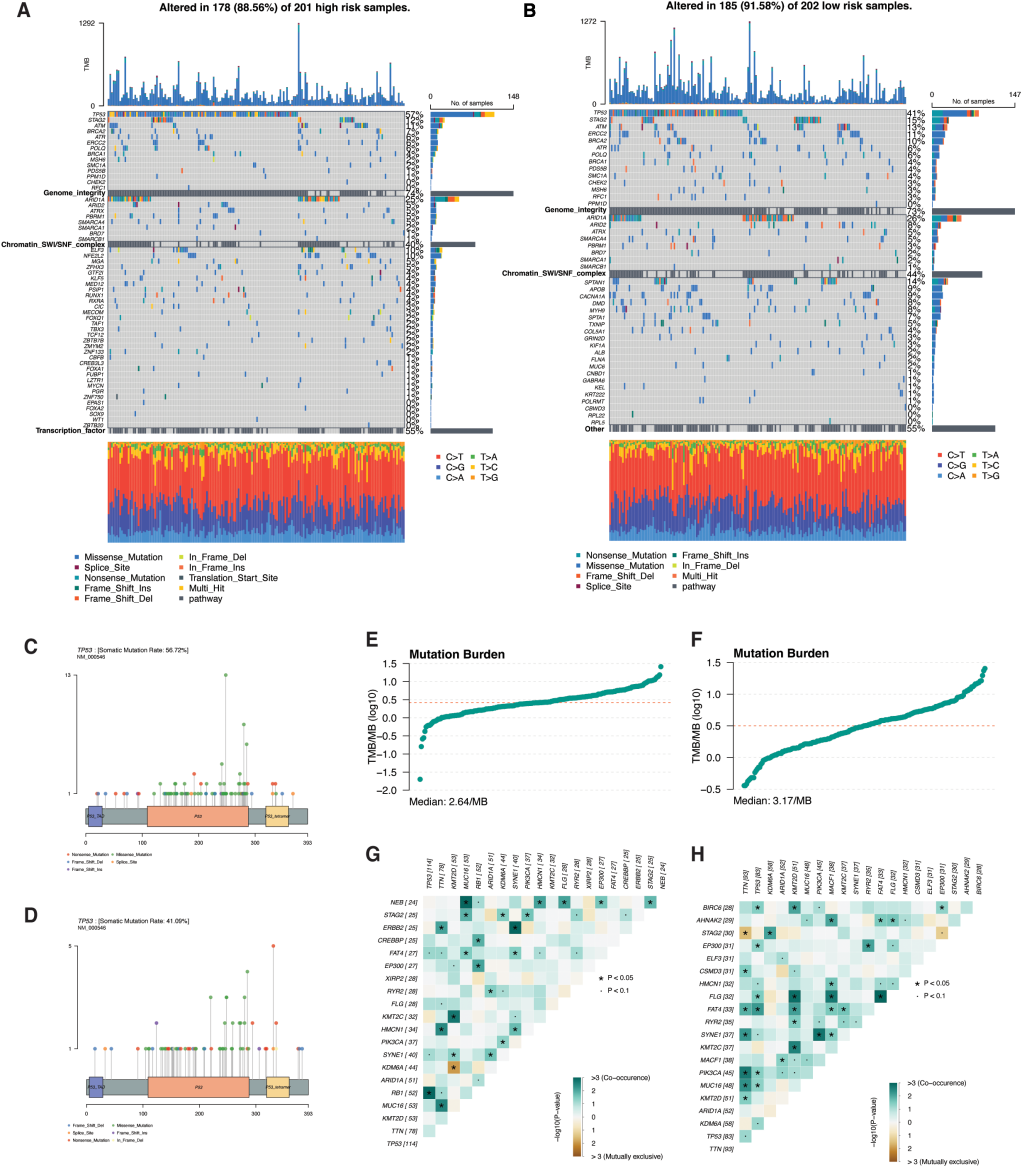
Mutation landscape of high- and low-risk PRG subtypes in bladder cancer. **(A, B)** Oncoplots showing the frequently mutated oncogenic pathways and their representative genes in the high-risk **(A)** and low-risk **(B)** PRG subtypes. **(C, D)** Lollipop plots displaying the distribution and frequency of TP53 mutations in the high-risk **(C)** and low-risk **(D)** groups. **(E, F)** Bar graphs comparing tumor mutation burden (TMB) between high-risk **(E)** and low-risk **(F)** subgroups. Tumor mutation burden was calculated as the number of somatic mutations per megabase of coding sequence (mut/Mb) **(G, H)** Somatic interaction plots illustrating co-occurring and mutually exclusive mutations in the high-risk **(G)** and low-risk **(H)** groups.

We conducted an in-depth analysis of genomic aberrations across the PRG subtypes to uncover key mutational patterns. The oncoplot visualizations highlight distinct mutation distributions between PRG subtypes (**Figures 4A, B**). The oncoplot of mutational pathways revealed three key altered pathways distinguishing bladder cancer risk groups. The genome integrity pathway, characterized by mutations in TP53 and STAG2, and the Chromatin SWI/SNF complex pathway, defined by ARID1A mutations, were observed across cohorts. Additionally, a transcription factor pathway driven by ELF3 and NFE2L2 mutations was exclusive to the high-risk subgroup, whereas the low-risk subgroup exhibited an “Other” pathway defined by mutations in SPTAN1, APOB, and CACNA1A. The lollipop plot illustrated a higher mutation rate in the high-risk subgroup (56.72%, max height 13) compared to the low-risk subgroup (41.09%, max height 5), with mutation hotspots clustering within key functional domains of TP53 (**Figures 4C, D**). Mutation burden analysis demonstrated that, despite the higher mutation rate in the high-risk group, the low-risk subgroup showed a slightly greater overall mutation burden (3.17 mut/Mb *vs*. 2.64 mut/Mb) (**Figures 4E, F**). This suggests that mutations in the low-risk group tend to affect larger genomic regions despite occurring in fewer patients. Somatic interaction analysis further revealed distinct co-occurrence patterns (**Figures 4G, H**). TP53 mutations frequently co-occurred with RB1, while TTN mutations predominantly co-occurred with MUC16. Notably, the low-risk subgroup exhibited more co-occurring partner genes for both TP53 and TTN than the high-risk subgroup, indicating a higher frequency and broader mutational diversity within low-risk tumors. Together, these results delineate distinct mutational landscapes and genomic interactions between bladder cancer risk groups, providing insights that may inform tailored therapeutic approaches.

### Transcriptomic differences between PRG subtypes

**Figure 5:**
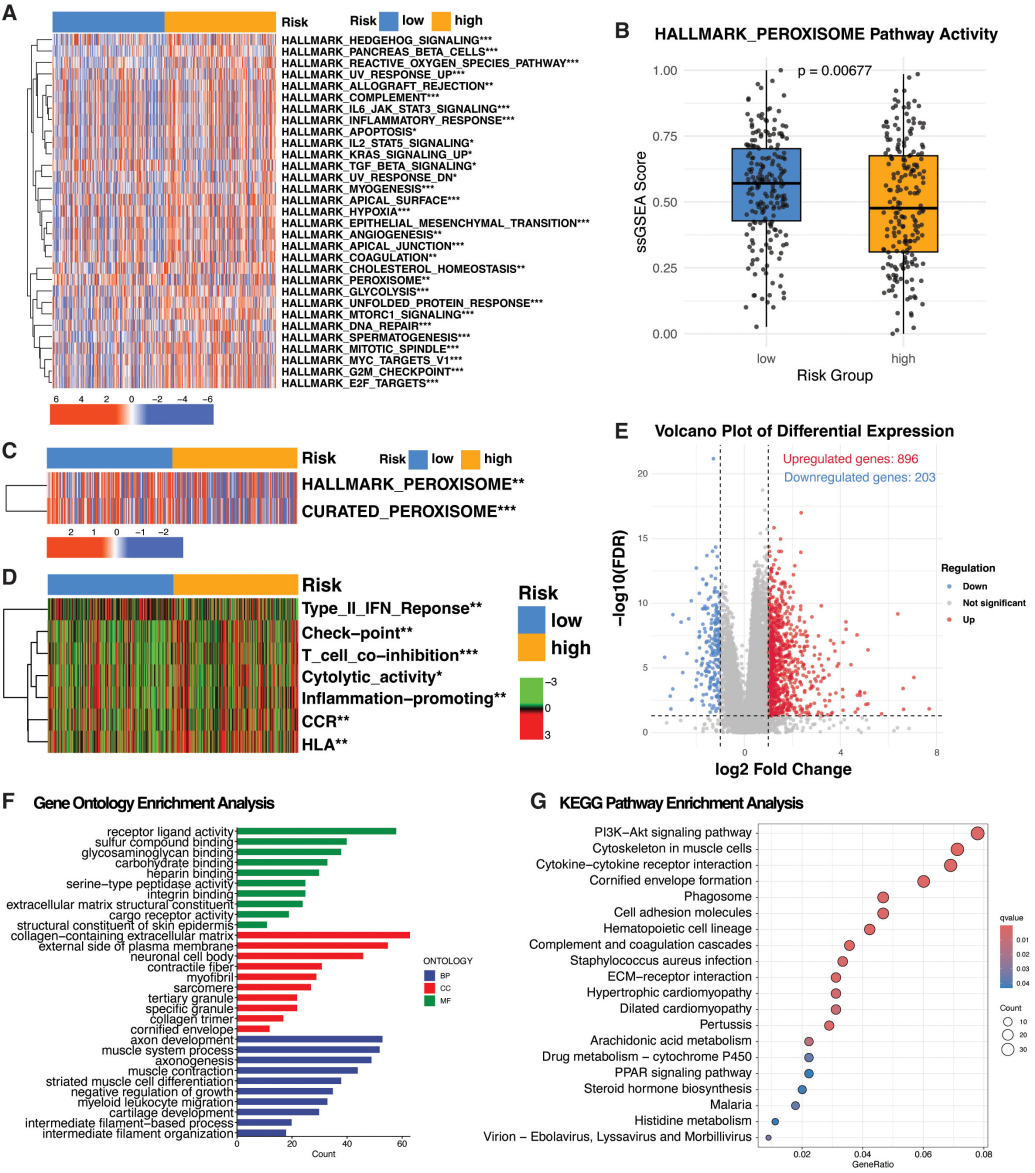
Transcriptomic Alterations Between PRG Subtypes in Bladder Cancer **(A)** Heatmap showing the differential activity of Cancer Hallmark pathways across PRG subtypes based on single-sample gene set enrichment analysis (ssGSEA). **(B)** Box plot comparing enrichment scores of the HALLMARK PEROXISOME pathway between high- and low-risk subgroups. **(C)** Heatmap displaying enrichment of the HALLMARK PEROXISOME and CURATED PEROXISOME pathways across PRG subtypes. **(D)** Heatmap showing enrichment scores of immune-related pathways among PRG subtypes. **(E)** Volcano plot showing differentially expressed analysis between PRG subtypes. **(F)** Bar plot illustrating Gene Ontology (GO) enrichment analysis of differentially expressed genes (DEGs) between PRG subtypes. **(G)** Bubble plot showing Kyoto Encyclopedia of Genes and Genomes (KEGG) pathway enrichment based on DEGs between PRG subtypes. *P < 0.05; **P < 0.01; ***P < 0.001; statistical significance determined using the Wilcoxon rank-sum test.

The identified PRG subtypes exhibited distinct clinical, molecular, and mutational characteristics. To further explore the underlying biological mechanisms differentiating these subtypes, we conducted a transcriptomic analysis to uncover key functional pathways. Pathway enrichment analysis revealed broad activation of hallmark oncogenic and stress-response pathways in the high-risk subgroup (**Figure 5A**). These included key cell cycle regulators (E2F targets, G2M checkpoint, MYC targets V1, mitotic spindle), DNA damage repair, hypoxia, EMT, angiogenesis, apoptosis, and immune-related signatures such as inflammatory response, IL6-JAK-STAT3 signaling, and allograft rejection, suggesting a more aggressive tumor phenotype. In contrast, the peroxisome pathway was among the few significantly upregulated in the low-risk subgroup, indicating a more regulated metabolic state (**Figures 5B, C**). As ACSL5 and XDH were the only PRGs associated with a protective prognostic effect, and ACSL5 is a key enzyme in long-chain fatty acid metabolism, ACSL5 may serve as a peroxisome-related biomarker of favorable prognosis in bladder cancer. Conversely, PRDX1, a peroxisomal antioxidant enzyme strongly expressed in the high-risk subgroup, may reflect heightened oxidative stress and survival signaling. Together, these results highlight distinct metabolic and stress-adaptive signaling between risk subgroups, with peroxisome regulation—particularly via ACSL5 and PRDX1—contributing to the biological divergence and prognostic differences observed.

Separately, several immune-related pathways showed distinct enrichment patterns between subgroups (**Figure 5D**). The low-risk subgroup was characterized by enrichment of the Type II interferon response, whereas the high-risk subgroup exhibited enrichment of HLA, CCR signaling, inflammation-promoting responses, T cell co-inhibition and checkpoint pathways, and cytolytic activity. These differences highlight subgroup-specific variations in immune regulation, surveillance, and inflammatory signaling.

GO and KEGG enrichment analyses based on differentially expressed genes (DEGs = 27; logFC ≥ 0.5, adjusted p-value < 0.05) further supported the biological divergence between subgroups (**Figures 5E–G**). GO terms highlighted enrichment in receptor–ligand interactions, extracellular matrix organization, and serine-type peptidase activity, suggesting differences in cell adhesion, matrix remodeling, and intercellular communication. Terms related to muscle structure and immune cell migration also indicated subgroup-specific variation in tissue and immune processes (**Figure 5F**). KEGG analysis identified significant enrichment in PPAR signaling, a key regulator of lipid metabolism and inflammation, along with ECM–receptor interaction, cytokine signaling, and steroid hormone biosynthesis. These findings underscore subgroup differences in metabolic regulation, immune signaling, and hormonal activity, aligning with observed transcriptional and functional divergence (**Figure 5G**).

### High-risk PRG subtype shows enhanced immune-stromal infiltration

**Figure 6:**
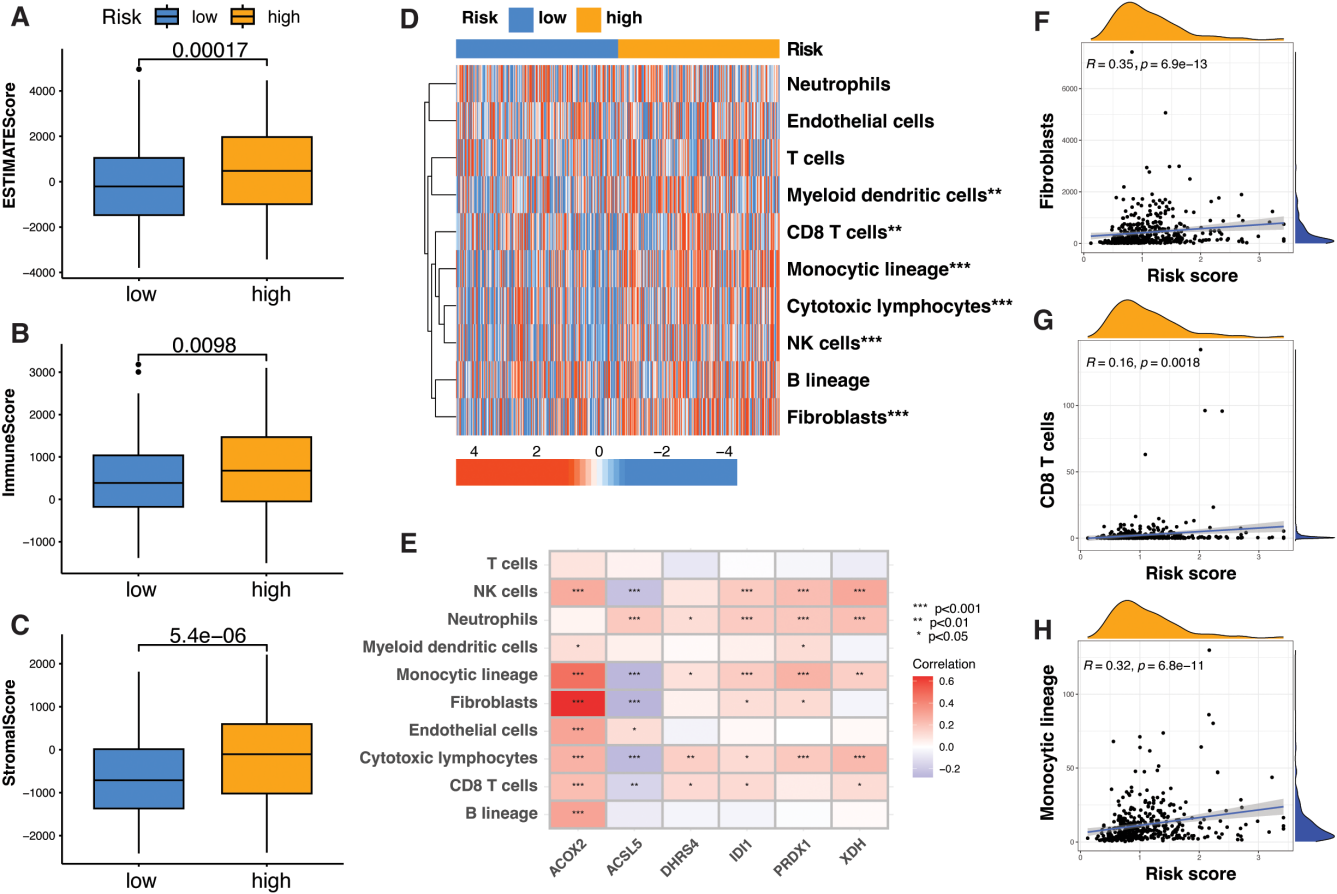
Tumor microenvironment characteristics associated with PRG subtypes. **(A–C)** Bar plots showing comparisons of ESTIMATE score **(A)**, immune score **(B)**, and stromal score **(C)** across PRG subtypes, indicating differences in the tumor microenvironment composition. **(D)** Heatmap depicting immune and stromal cell infiltration across PRG subtypes based on MCP-counter analysis. **(E)** Heatmap illustrating Spearman correlation between individual PRG signature genes and immune cell infiltration. **(F–H)** Scatter plots showing Spearman correlation between PRG riskScore and the infiltration levels of fibroblasts **(F)**, **(F–H)** Scatter plots showing Spearman correlation between PRG riskScore and the infiltration levels of fibroblasts **(F)**, CD8+ T cells **(G)**, and monocytic lineage cells **(H)**. *P < 0.05; **P < 0.01; ***P < 0.001; statistical significance assessed using the Wilcoxon rank-sum test and Spearman’s correlation, as appropriate.

To further understand the tumor microenvironment (TME) differences between the two PRG subtypes, we evaluated the immune and stromal components using the ESTIMATE (Estimation of STromal and Immune cells in MAlignant Tumor tissues using Expression data) algorithm, which infers stromal and immune cell presence based on gene expression data. This analysis revealed a significantly enriched tumor microenvironment in the high-risk subgroup, characterized by higher immune- and stromal-related signature scores, as reflected by elevated ImmuneScore and StromalScore values derived from the ESTIMATE algorithm (**Figures 6A–C**).

To precisely characterize cellular composition, we applied the MCP-counter (Microenvironment Cell Populations counter) algorithm, which identified a notable increase in monocytic lineage cells, natural killer (NK) cells, dendritic cells of monocytic lineage, cytotoxic T cells, CD8+ T cells, and fibroblasts within the high-risk subgroup (**Figure 6D**). At the gene level, ACOX2 and PRDX1 exhibited positive correlations with immune and stromal cell abundance scores, whereas ACSL5 showed an overall negative correlation trend (**Figure 5E**). Notably, the most pronounced correlations were observed with monocytic lineage cells and fibroblasts, while associations with other immune cell types were comparatively modest. In addition, the PRG risk score showed positive correlations with the enrichment levels of multiple immune and stromal cell populations, particularly fibroblasts and monocytic lineage cells, as illustrated for representative cell types in **Figures 6F–H**.

### PRDX1 and ACSL5 as key biomarkers distinguishing PRG-based BLCA subtypes

**Figure 7:**
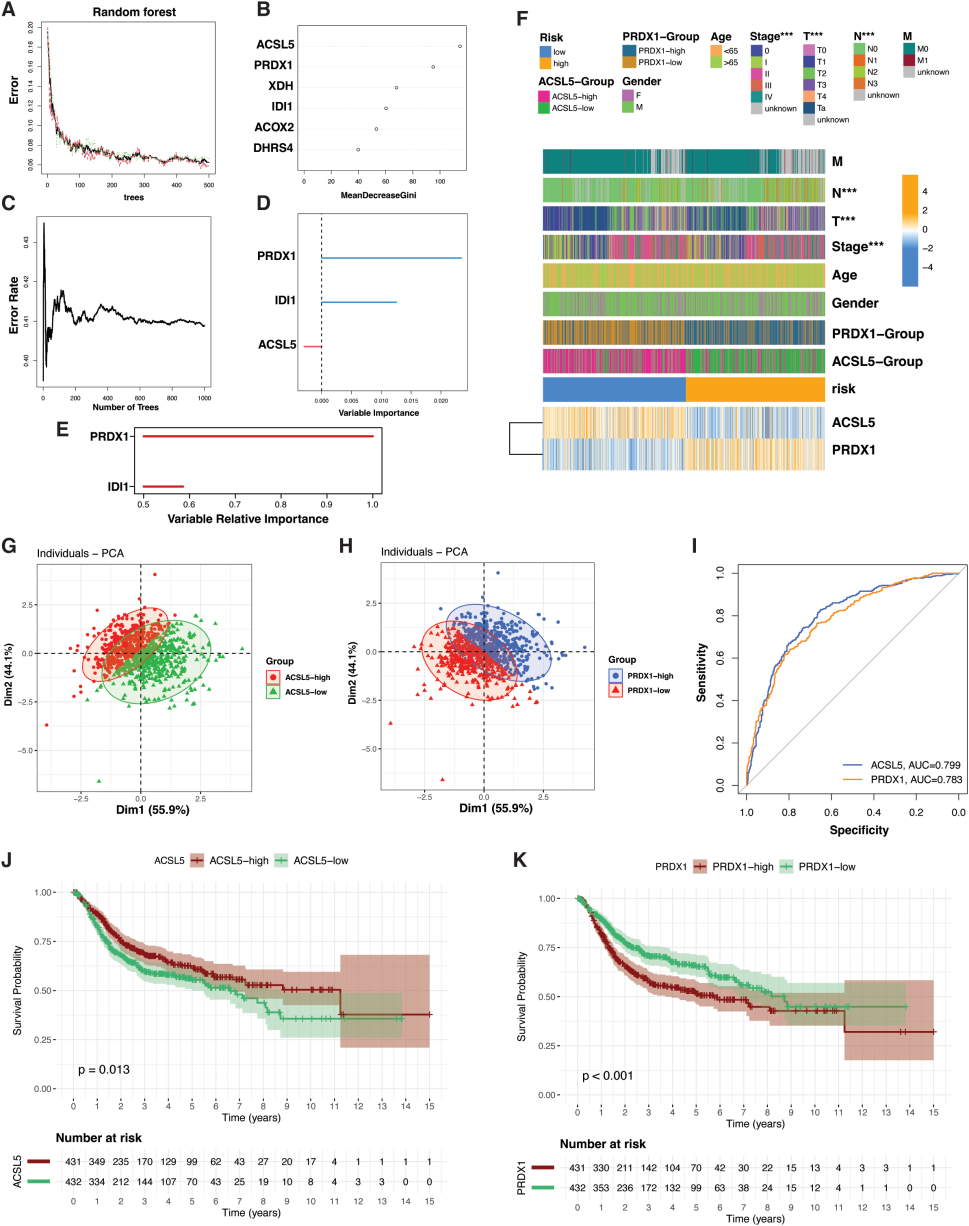
Identification of PRDX1 and ACSL5 as diagnostic and prognostic biomarkers in PRG-based bladder cancer subtypes. **(A)** Classification error rate plot from the diagnostic random forest model showing performance with increasing numbers of trees. **(B)** Feature importance ranked by MeanDecreaseGini, highlighting ACSL5 and PRDX1 as top diagnostic genes. **(C)** Error rate plot from the random survival forest model evaluating prognostic relevance. **(D)** Out-of-bag variable importance scores from the survival forest model identifying PRDX1 as the most significant prognostic marker. **(E)** Bar plot displaying prognostic variables with relative importance > 0.5. **(F)** Heatmap showing clinical and molecular characteristics of BLCA patients stratified by median expression of PRDX1 and ACSL5. ***P < 0.001; statistical significance assessed using the Chi-square test. **(G, H)** PCA plots illustrating separation of patient subgroups based on PRDX1 **(G)** and ACSL5 **(H)** expression, with PC1 explaining 55.9% of the variance. **(I)** ROC curves evaluating the classification performance of PRDX1 and ACSL5 between high- and low-risk subgroups. **(J, K)** Kaplan–Meier survival curves comparing overall survival between high *vs*. low expression groups for PRDX1 **(J)** and ACSL5 **(K)**.

To identify robust biomarkers that distinguish PRG-based subtypes in BLCA, we employed a random forest classifier. Among the diagnostic feature genes, PRDX1 and ACSL5 emerged as the top two candidates based on the MeanDecreaseGini index, indicating their strong contribution to subtype classification (**Figures 7A, B**). To evaluate their prognostic relevance, we conducted univariate Cox regression and random survival forest analyses. Notably, PRDX1 showed the highest variable importance score in the survival forest analysis, followed closely by IDI1 and ACSL5 (**Figures 7C–E**), underscoring their association with overall survival within PRG-defined risk groups. Next, we stratified patients according to the median expression levels of PRDX1 and ACSL5. Heatmap visualization revealed strong concordance between gene-based classification and established risk subgroups, as well as key clinical features (**Figure 7F**). PCA further supported this stratification, with the first PC1 explaining 55.9% of the total variance between subgroups (**Figures 7G, H**). Diagnostic performance was assessed using ROC analysis, where ACSL5 achieved an AUC of 0.799 and PRDX1 an AUC of 0.783, highlighting their effectiveness in differentiating PRG-based subtypes (**Figure 7I**). Moreover, Kaplan–Meier survival analysis revealed a significant difference in overall survival between high and low ACSL5 expression groups (p = 0.013), while PRDX1 showed even stronger prognostic value, with an inverse survival trend and highly significant difference (p < 0.001) (**Figures 7J, K**). Collectively, these results position PRDX1 and ACSL5 as representative diagnostic and prognostic biomarkers for PRG-based molecular stratification in BLCA, with potential utility in improving patient risk assessment and personalized treatment strategies.

### Experimental validation of PRG risk genes in bladder cancer cell proliferation

**Figure 8:**
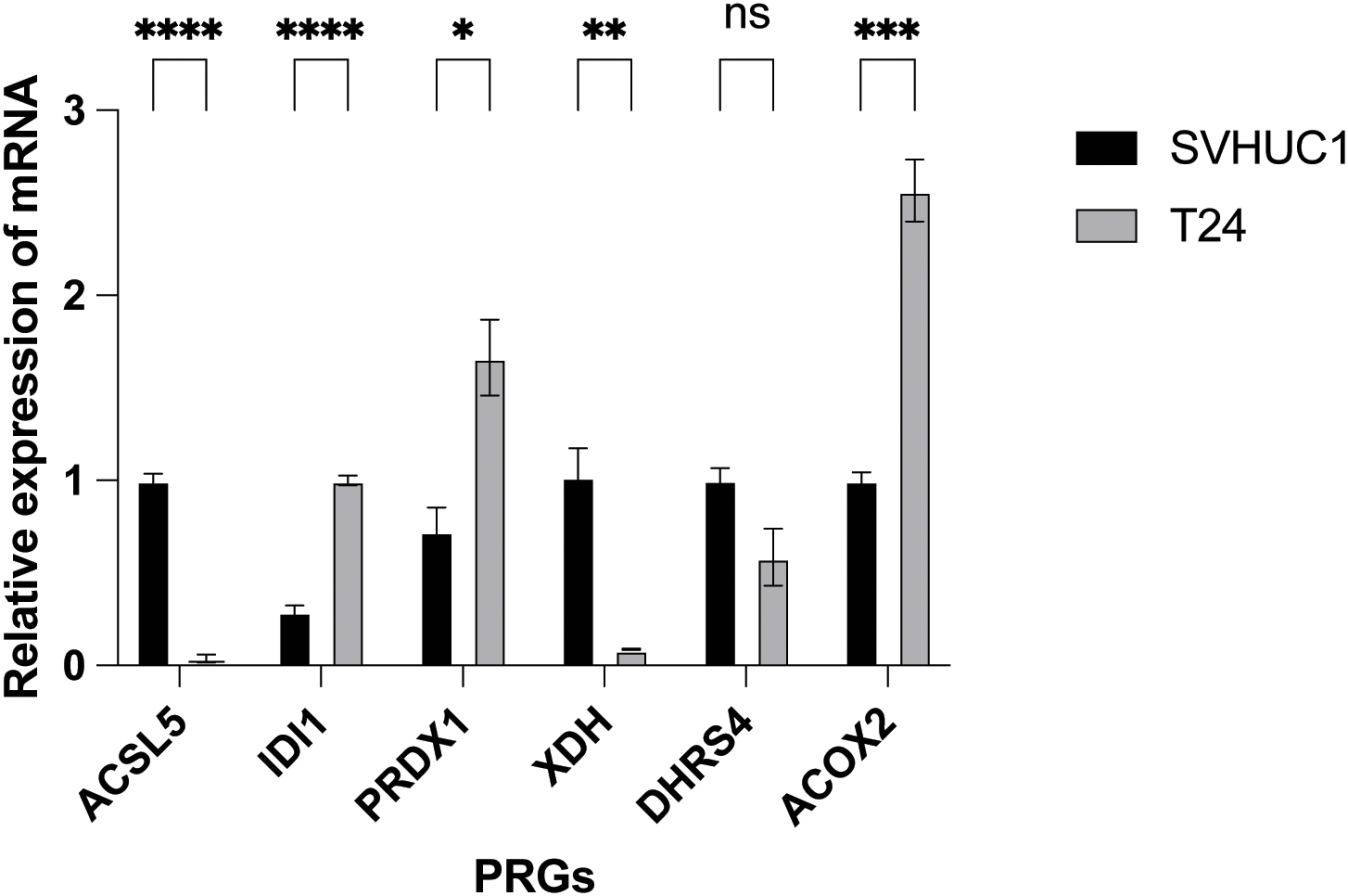
Experimental validation of PRGs in bladder cancer cells. Relative mRNA expression levels of PRG risk genes in normal bladder epithelial cell line XVHUC1 and human bladder cancer cell line T24 as assessed by qualitative PCR. The data represent the mean ± SEM (standard error of mean) of n=3 independent experiments (independent biological replicas) for each condition. Two-tailed unpaired T-test; *P<0.05; **P < 0.01; ***P < 0.001; ****P < 0.0001; ns, not significant.

We experimentally assessed the mRNA expression levels of candidate PRG risk genes in the bladder cancer cell line T24, compared to the normal bladder epithelial cell line XVHUC1. The analysis revealed that high risk PRG genes were significantly upregulated in T24 cells relative to XVHUC1 cells. ACSL5 showed significant downregulation while XDH did not show any significant change in expression (**Figure 8**).

### Drug-gene enrichment identifies erythorbic acid as a lead compound

**Figure 9:**
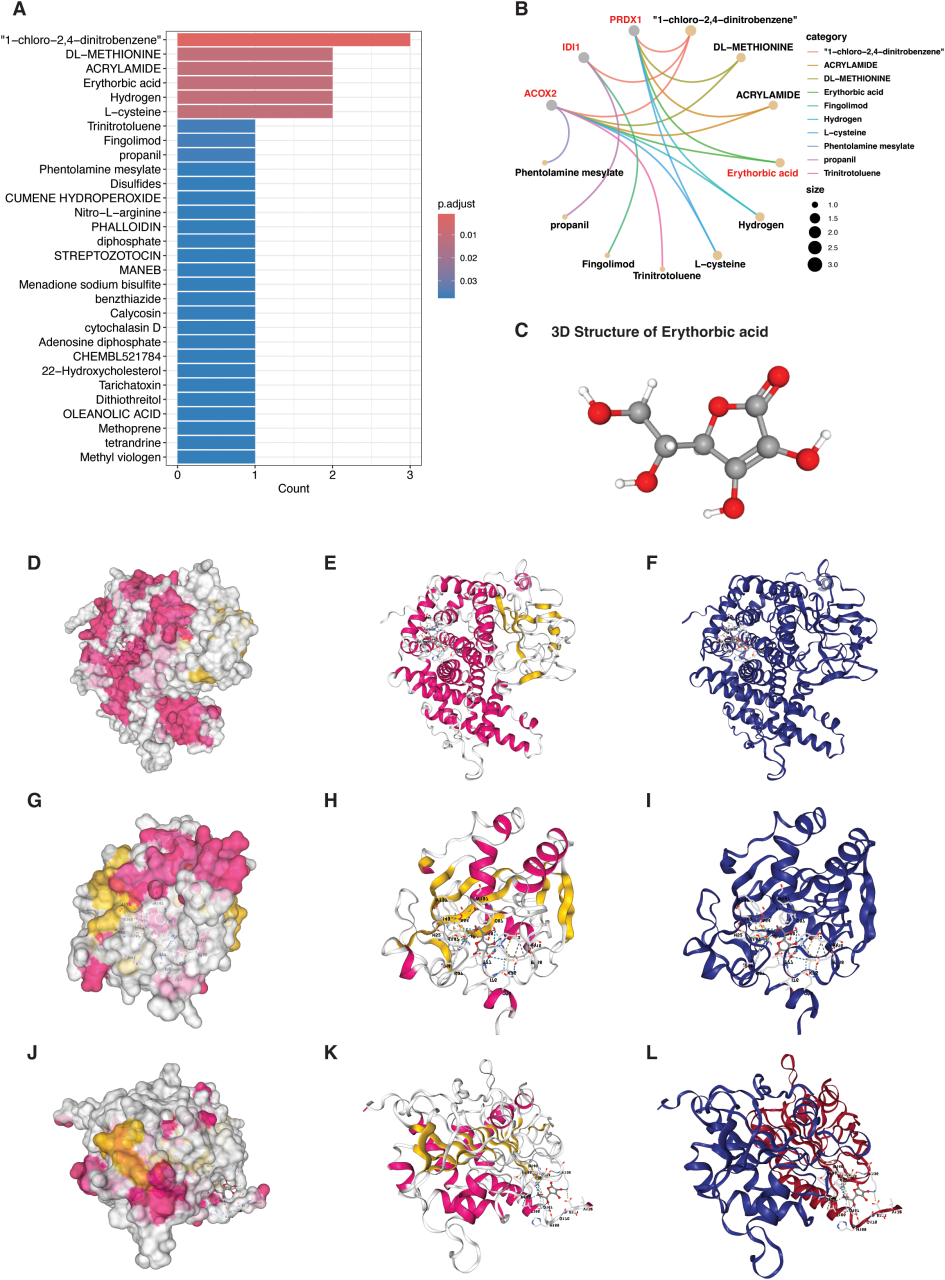
Drug–gene enrichment and docking analysis of erythorbic acid targeting PRDX1 **(A)** Bar plot of top-ranked compounds identified by drug–gene enrichment analysis using DSigDB. **(B)** Network diagram showing predicted interactions between drugs and PRG hub genes. **(C)** Structures of erythorbic acid (PubChem) used for docking analysis. **(D–F)** Erythorbic acid binding to the largest catalytic pocket of ACOX2, displayed in three receptor representations: **(D)** Surface (Color: Secondary), **(E)** Cartoon (Color: Secondary), and **(F)** Cartoon (Color: Chain). **(G–I)** Binding interaction of erythorbic acid within the Mn²^+^-coordinated catalytic site of IDI1, shown in **(G)** Surface (Color: Secondary), **(H)** Cartoon (Color: Secondary), and **(I)** Cartoon (Color: Chain) views. **(J–L)** Docking of erythorbic acid to the peroxidatic cysteine region (Pocket 2) of PRDX1, visualized as **(J)** Surface (Color: Secondary), **(K)** Cartoon (Color: Secondary), and **(L)** Cartoon (Color: Chain).

Drug-gene interaction enrichment analysis using the DSigDB database identified several compounds significantly associated with the three high-risk peroxisome-related genes (ACOX2, IDI1, and PRDX1) (**Figure 9A**). Among the top statistically significant hits, 1-chloro-2,4-dinitrobenzene exhibited the lowest p-value (9.29 × 10^-6^) and interacted with all three target genes, followed by DL-methionine, acrylamide, erythorbic acid, hydrogen, and L-cysteine, each associated with subsets of ACOX2 and PRDX1 (**Figure 9B**). However, given the known toxic or nonspecific reactivity of compounds such as 1-chloro-2,4-dinitrobenzene and acrylamide, erythorbic acid was prioritized as the most biologically relevant and pharmacologically safe candidate for subsequent molecular docking. Erythorbic acid demonstrated significant enrichment (p = 5.3 × 10^-4^; FDR = 0.0107) and shared strong predicted associations with both ACOX2 and PRDX1, two enzymes upregulated in the high-risk molecular subgroup of bladder cancer. These results supported the selection of erythorbic acid for in-depth docking and structural interaction analysis.

### Molecular docking reveals stable binding of erythorbic acid to multiple high-risk PRG targets

To investigate the binding interaction of erythorbic acid with PRGs, we first retrieved the 3D chemical structure of erythorbic acid from the PubChem database as shown in **Figure 9C**. Protein 3D structures for ACOX2, IDI1 and PRDX1 were also obtained and were then used as input for docking analysis via CB-Dock2, a cavity-detection-based blind docking platform (**Figures 9D–F**). Molecular docking of erythorbic acid with ACOX2, IDI1, and PRDX1 revealed stable and energetically favorable binding within functionally important regions of all three proteins (**Table 2**; **Figures 9D–L**).

**Table 2:** Docking Parameters and Catalytic Pocket Interactions of Erythorbic Acid with ACOX2, IDI1, and PRDX1.

Target protein	Pocket ID	Volume (Å³)	Binding center (x, y, z)	Vina Score (kcal/mol)	Key contact residues	Functional annotation / interpretation
**ACOX2**	**1**	**13 116**	(–2.37, –8.70, 6.81)	**–6.2**	THR37, ASP41, ARG50, TYR105, GLU437, PHE627, ASP628	Largest catalytic pocket adjacent to FAD-binding site; predicted primary binding cavity
	4	567	(16.16, –12.77, –19.84)	–5.2	HIS6, ARG7, GLN17, TYR641	Surface pocket; lower affinity
**IDI1**	**1**	**292**	(13.22, 10.52, 37.69)	**–6.6**	HIS41, HIS52, CYS87, ARG112, TYR137, TRP197	Mn²⁺-coordinated isomerase pocket; catalytically active region
	3	140	(30.28, 9.21, 47.83)	–5.3	ASN28, GLU98, TYR131, HIS222	Peripheral hydrophilic groove
**PRDX1**	**2**	**249**	(–19.94, 68.77, 8.23)	**–5.4**	LYS37, TYR38, LEU69, ASN70, ASP134, PHE165	Peroxidatic cysteine region; redox-active catalytic pocket
	1	801	(–9.39, 66.64, 24.15)	–4.9	ASP115, TYR116, LEU119, PHE127	Dimer interface; shallow binding

The pocket with the lowest docking energy and catalytically relevant residues for each target is indicated in bold.

For ACOX2, erythorbic acid occupied the enzyme’s largest FAD-adjacent pocket (volume = 13,116 Å³) with a binding energy of –6.2 kcal/mol (**Table 2**; **Figures 7D–F**). Key interacting residues included ARG50, TYR105, GLU437, and ASP628, consistent with the catalytic environment responsible for β-oxidation. This interaction suggests that erythorbic acid may modulate peroxisomal lipid oxidation and ROS production. In IDI1, erythorbic acid exhibited the strongest affinity (–6.6 kcal/mol) within the Mn²^+^-coordinated catalytic pocket, interacting with HIS41, HIS52, CYS87, and TYR137 (**Table 2**; **Figures 9G–I**). This site corresponds to the isomerase’s active domain involved in the mevalonate pathway, implicating erythorbic acid as a potential modulator of isoprenoid biosynthesis and redox coupling in tumor metabolism. For PRDX1, the compound bound stably near the peroxidatic cysteine region (Pocket 2; volume = 249 Å³; –5.4 kcal/mol), contacting residues LYS37, TYR38, LEU69, ASN70, ASP134, and PHE165 adjacent to Cys52, the active thiol responsible for peroxide reduction (**Table 2**; **Figures 7J-L**). This interaction indicates potential interference with PRDX1’s antioxidant function, which may reduce the redox resilience of high-risk tumor cells.

Overall, erythorbic acid demonstrated multi-target binding across metabolic and antioxidant PRG hubs, supporting its potential as a broad-spectrum peroxisomal modulator for high-risk bladder cancer subtypes.

### Erythorbic acid differentially reduces viability of bladder cancer and normal urothelial cells

**Figure 10:**
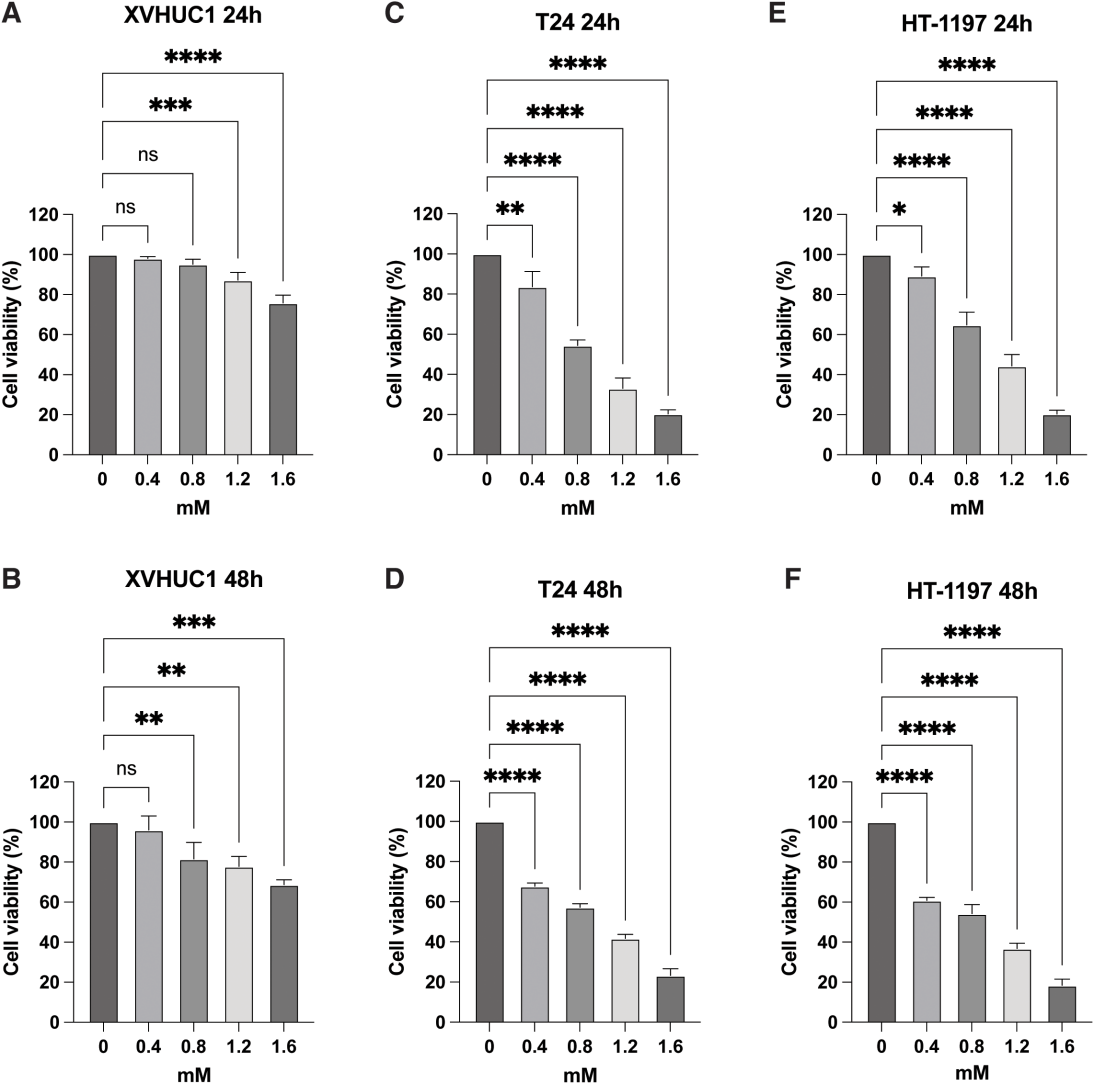
Erythorbic acid differentially reduces cell viability in normal urothelial and bladder cancer cell lines in a dose- and time-dependent manner. XV-HUC-1 normal urothelial cells **(A, B)**, T24 bladder cancer cells **(C, D)**, and HT-1197 bladder cancer cells **(E, F)** were treated with increasing concentrations of erythorbic acid (0, 0.4, 0.8, 1.2, and 1.6 mM). Cell viability was assessed using the CCK-8 assay at 24 h **(A, C, E)** and 48 h **(B, D, F)** following treatment. Absorbance at 450 nm was normalized to untreated controls (0 mM), which were set to 100% viability. Data are presented as mean ± SD from three independent experiments (n = 3). Statistical significance relative to control was determined by one-way ANOVA followed by appropriate *post hoc* testing and is indicated as *p < 0.05, **p < 0.01, ***p < 0.001, and ****p < 0.0001.

To evaluate the effect of erythorbic acid on bladder cancer cell viability, CCK-8 assays were performed in the bladder cancer cell lines T24 and HT-1197, with XV-HUC-1 normal urothelial cells included as a non-malignant comparator. Cells were treated with increasing concentrations of erythorbic acid and assessed at 24 h and 48 h. Erythorbic acid induced a dose- and time-dependent reduction in cell viability in both T24 and HT-1197 cells relative to untreated controls, with progressively greater decreases observed at higher concentrations and longer exposure durations. In contrast, XV-HUC-1 cells exhibited comparatively limited sensitivity, maintaining near-baseline viability at 0.4–0.8 mM, while higher concentrations resulted in only a moderate reduction, with viability remaining at or above approximately 80%, a level commonly regarded as indicative of minimal cytotoxic effects in *in vitro* assays of normal epithelial cells (19) (**Figure 10**).

## Discussion

Bladder cancer remains a highly heterogeneous malignancy, with considerable variability in clinical outcomes and therapeutic responses. In this study, we identified two distinct risk subgroups of BLCA patients based on the prognostic relevance of PRGs. These subgroups not only demonstrated divergent prognostic outcomes but also exhibited significant differences in their mutational profiles, activation of oncogenic pathways, and TME composition. Notably, the high-risk subgroup was characterized by mutations in transcription factors and marked upregulation of cancer hallmark pathways. This group also displayed a TME enriched with both immune and stromal components, suggesting a more complex and potentially immunosuppressive microenvironment. Intriguingly, despite the upregulation of four out of the six PRG signature genes in the high-risk subgroup, overall peroxisome enrichment was predominantly associated with the low-risk subgroup. Through diagnostic and survival-based random forest models, PRDX1 and ACSL5 emerged as key discriminatory biomarkers reflective of peroxisome activity. PRDX1 was linked with the high-risk phenotype, while ACSL5 was associated with the low-risk subgroup and favorable prognosis. Furthermore, erythorbic acid was identified as a promising candidate compound capable of modulating three out of the six PRG signature genes, including PRDX1. Molecular docking analyses supported the potential interaction between erythorbic acid and PRDX1, reinforcing its therapeutic relevance. Taken together, our findings highlight the prognostic and therapeutic implications of PRG-based stratification in BLCA, offering novel insights into molecular subtypes and potential avenues for targeted intervention.

In our analysis of bladder cancer cohorts, two distinct molecular subgroups were identified based on the expression profiles of key peroxisome-related genes. One subgroup, associated with better prognosis, showed elevated expression of ACSL5, an enzyme that activates long-chain fatty acids for lipid metabolism. ACSL5 is localized not only to the endoplasmic reticulum and mitochondrial outer membrane but also to peroxisomes, where it catalyzes the formation of fatty acyl-CoAs, critical intermediates in lipid metabolism (20). The enhanced peroxisomal fatty acid metabolism associated with ACSL5 may support tumor-suppressive processes and improved therapeutic responses. Notably, high ACSL5 expression has been linked to favorable prognosis in breast cancer, where it is associated with reduced recurrence and better survival outcomes (21). Although the prognostic significance of ACSL5 in other malignancies—such as colorectal and pancreatic cancers—is less consistent, its expression has been implicated in cancer metabolism and disease progression (21–23). These findings suggest that ACSL5 plays a context-dependent role in cancer biology, and in the case of bladder cancer, its upregulation may define a metabolically distinct and clinically favorable subtype.

In contrast, the second subgroup exhibits elevated expression of PRDX1, a critical antioxidant enzyme that reduces cellular peroxide levels and protects cells from oxidative stress. High PRDX1 expression correlates with poor prognosis, potentially due to its role in promoting tumor cell survival and resistance to oxidative damage, which may impair therapy effectiveness. Notably, this subgroup also demonstrated a significantly higher mutation frequency in NFE2L2 (NRF2), a master regulator of the antioxidant response (24). The co-occurrence of PRDX1 overexpression and NFE2L2 mutation suggests a synergistic activation of redox-protective pathways, allowing tumor cells to evade oxidative stress and therapeutic pressure.

PRDX1 plays diverse oncogenic roles across multiple cancer types. In breast cancer, PRDX1 enhances NF-κB activity and inhibits apoptosis, contributing to treatment resistance and recurrence (25). In esophageal cancer, it promotes tumorigenesis via activation of the mTOR/p70S6K pathway and suppression of ROS-induced cytotoxicity (26). In lung cancer, PRDX1 drives epithelial–mesenchymal transition (EMT) and metastasis through TGF-β1 signaling and protects against apoptosis via modulation of ROS/JNK and ERK pathways (27, 28). In prostate cancer, PRDX1 supports tumor angiogenesis and androgen receptor signaling by activating the TLR4–NF-κB–HIF-1α axis (29). In pancreatic cancer, it facilitates invasion by regulating p38MAPK and promoting membrane protrusion formation (30). In cervical cancer, PRDX1 knockdown enhances chemosensitivity to ROS-generating agents (31). In bladder cancer, PRDX1 contributes to tumor growth via NF-κB activation and protects against ROS-based therapeutic stress (32). Its overexpression has also been documented in gallbladder, cholangiocarcinoma, and liver cancers, indicating a broad role in oxidative stress adaptation, though mechanisms in these contexts remain less defined (33–35). Together, these findings implicate the PRDX1–NFE2L2 axis as a central mechanism of redox adaptation and therapeutic resistance in cancer.

The molecular docking analysis revealed that erythorbic acid, an antioxidant stereoisomer of ascorbic acid widely used in pharmaceuticals and food preservation, interacts with three high-risk peroxisome-related enzymes (ACOX2, IDI1, and PRDX1) with binding affinities ranging from –5.4 to –6.6 kcal/mol. Erythorbic acid exhibits strong redox-modulating and ROS-scavenging properties but with lower cytotoxicity than ascorbic acid, making it a potential candidate for targeting oxidative and metabolic imbalance in cancer cells (36–38). Given that the high-risk PRG subgroup in bladder cancer is characterized by elevated oxidative stress and altered lipid metabolism, erythorbic acid’s dual antioxidant and metabolic regulatory roles make it a rational therapeutic scaffold for further evaluation.

For ACOX2, erythorbic acid occupied the enzyme’s largest catalytic cavity (–6.2 kcal/mol), forming interactions with residues ARG50, TYR105, GLU437, and ASP628 near the FAD-binding site. ACOX2 catalyzes the first step of peroxisomal β-oxidation, coupling fatty-acid catabolism to hydrogen peroxide generation (39). ACOX2 dysregulation has been implicated in altered lipid utilization, peroxisomal ROS accumulation, and tumor progression in hepatocellular, and prostate cancer (40, 41). In addition to roles in lipid catabolism and ROS generation, ACOX2 has been shown to destabilize the MRE11-RAD50-NBS1 complex and activate cGAS-STING-mediated immune responses in ccRCC, further underscoring its multifaceted tumor-suppressive potential (42). The observed binding indicates that erythorbic acid may stabilize or buffer excessive ROS production without fully inhibiting β-oxidation, aligning with its role as a mild redox regulator.

Docking with IDI1 showed the strongest affinity (–6.6 kcal/mol) within the Mn²^+^-coordinated catalytic pocket, involving key residues HIS41, HIS52, CYS87, and TYR137.

IDI1 catalyzes a critical step in the mevalonate pathway, producing isoprenoid intermediates essential for cholesterol biosynthesis and prenylation of oncogenic proteins. Aberrant activation of this pathway enhances proliferation, invasion, and therapeutic resistance in various tumors (43, 44). The predicted binding of erythorbic acid in this region suggests it could attenuate mevalonate-derived lipid signaling, potentially reducing metabolic plasticity in aggressive bladder cancer cells.

For PRDX1, erythorbic acid exhibited a moderate but specific binding affinity (-5.4 kcal/mol) within the peroxidatic cysteine region (Cys52), involving residues ASP134, PHE165, and GLU171, which are functionally associated with catalytic redox regulation (45–47).

PRDX1 plays a crucial role in detoxifying hydrogen peroxide and maintaining redox homeostasis, and its overexpression has been linked to enhanced tumor survival and therapy resistance under oxidative stress conditions. The predicted interaction suggests that erythorbic acid may partially interfere with PRDX1’s peroxidase activity, thereby increasing intracellular ROS and sensitizing cancer cells to oxidative stress. This moderate yet specific binding supports a reversible modulatory mechanism, consistent with erythorbic acid’s antioxidant properties, and indicates that it could act as a fine-tuned regulator of peroxiredoxin-mediated redox balance in PRDX1-overexpressing bladder cancers (48). Collectively, these results indicate that erythorbic acid engages a network of redox-sensitive peroxisomal enzymes, spanning both antioxidant defense (PRDX1) and oxidative metabolism (ACOX2, IDI1). Such multi-target activity suggests erythorbic acid functions as a redox-metabolic modulator, capable of restoring oxidative balance and mitigating hyperactive metabolic signaling within the high-risk PRG subgroup of bladder cancer. Moreover, erythorbic acid significantly reduced bladder cancer cell viability *in vitro*, supporting the bioinformatic association between peroxisome-related pathways and tumor behavior. In contrast, normal urothelial XV-HUC-1 cells maintained near-baseline viability at 0.4-0.8 mM and remained at or above approximately 80% viability at higher concentrations, indicating minimal cytotoxic effects (19). While these *in vitro* findings suggest differential cellular responses to erythorbic acid, further validation in *in vivo* bladder cancer models is required to determine its therapeutic relevance and safety, as well as to elucidate the underlying mechanisms linking erythorbic acid to peroxisome-associated metabolic pathways.

The role of peroxisomes in cancer has been explored through bioinformatic analyses in various malignancies, including hepatocellular carcinoma (HCC), lower-grade glioma (LGG), renal clear cell carcinoma (RCC), breast cancer, and colorectal cancer (49–53). Among the genes identified in our bladder cancer PRG signature, only ACSL5 has previously been reported as a risk-related PRG in LGG. This minimal overlap underscores the distinct peroxisomal regulatory mechanisms operative in bladder cancer, suggesting a cancer-type-specific involvement of PRGs. Such variability highlights the heterogeneous roles of peroxisomes across tumor types, shaped by unique metabolic and immune microenvironments, and emphasizes the importance of context-specific biomarker development. Moreover, although this study included functional validation using CCK-8 assays, the absence of in-depth mechanistic investigations remains a limitation, and further studies are needed to clarify the molecular pathways through which erythorbic acid and peroxisome-related genes affect bladder cancer progression. Finally, while ComBat was applied to reduce batch effects between TCGA RNA-seq and GEO microarray datasets, cross-platform harmonization cannot fully eliminate technology-driven differences; therefore, integrative analyses were based on relative expression patterns and should be interpreted with appropriate caution (54, 55).

## Conclusions

This study establishes peroxisome-related genes as key determinants of metabolic and redox heterogeneity in bladder cancer. High-risk tumors were defined by upregulation of *PRDX1*, *ACOX2*, and *IDI1*, reflecting enhanced oxidative stress tolerance and lipid metabolic reprogramming. The identification of erythorbic acid as a safe, multi-target modulator capable of binding these enzymes highlights a potential therapeutic strategy aimed at rebalancing peroxisomal redox–metabolic activity. Collectively, these findings position peroxisomal dysfunction as a hallmark of aggressive bladder cancer and propose erythorbic acid as a promising scaffold for redox-based therapeutic intervention.

## Data Availability

The original contributions presented in the study are included in the article/**Supplementary Material**. Further inquiries can be directed to the corresponding author.
