# Supplementary material for: Peroxisomal activity drives aggressive bladder cancer phenotypes and reveals erythorbic acid as a potential therapeutic modulator
Source: Front Oncol. 2026 Mar 4;16:1734437. doi: 10.3389/fonc.2026.1734437 (PMC12995624; doi:10.3389/fonc.2026.1734437)
Supplement: Supplementary file 1 [file DataSheet1.docx]

**Supplementary Data**

**Supplementary Code S1: TCGA-BLCA FPKM retrieval**

## Libraries

library(TCGAbiolinks)

library(SummarizedExperiment)

## Query TCGA-BLCA gene expression data

query_TCGA_BLCA <- GDCquery(

project = "TCGA-BLCA",

data.category = "Transcriptome Profiling",

data.type = "Gene Expression Quantification",

workflow.type = "STAR - Counts",

access = "open"

)

## Download data

GDCdownload(query_TCGA_BLCA)

## Prepare SummarizedExperiment

tcga_blca_se <- GDCprepare(query_TCGA_BLCA, summarizedExperiment = TRUE)

## Retrieve FPKM (unstranded)

fpkm_unstranded <- assay(tcga_blca_se, "fpkm_unstranded")

**Supplementary Code S2: Gene ID to Gene Symbol Conversion (Perl)**

use strict;

#use warnings;

my $gtfFile="human.gtf";

my $expFile="mRNAmatrix.txt";

my $outFile="symbol.txt";

my %hash=();

open(RF,"$gtfFile") or die $!;

while(my $line=<RF>)

{

chomp($line);

if($line=~/gene_id \"(.+?)\"\;.+gene_name "(.+?)"\;.+gene_biotype \"(.+?)\"\;/)

{

$hash{$1}=$2;

}

}

close(RF);

open(RF,"$expFile") or die $!;

open(WF,">$outFile") or die $!;

while(my $line=<RF>)

{

if($.==1)

{

print WF $line;

next;

}

chomp($line);

my @arr=split(/\t/,$line);

$arr[0]=~s/(.+)\..+/$1/g;

if(exists $hash{$arr[0]})

{

$arr[0]=$hash{$arr[0]};

print WF join("\t",@arr) . "\n";

}

}

close(WF);

close(RF);

**Supplementary Code S3: Cross-platform integration of TCGA (RNA-seq) and GEO (microarray) expression data using ComBat**

library(limma)

library(sva)

# Input files

tcgaExpFile <- "tcga.txt" # TCGA expression matrix (gene symbols in first column)

geoExpFile <- "geo.txt" # GEO expression matrix (gene symbols in first column)

geneFile <- "PRGs.txt" # PRG list (gene symbols in first column)

## 1) Read and preprocess TCGA

rt <- read.table(tcgaExpFile, header = TRUE, sep = "\t", check.names = FALSE)

rt <- as.matrix(rt)

rownames(rt) <- rt[, 1]

exp <- rt[, 2:ncol(rt)]

dimnames <- list(rownames(exp), colnames(exp))

tcga <- matrix(as.numeric(as.matrix(exp)),

nrow = nrow(exp),

dimnames = dimnames)

# Collapse duplicated gene symbols (if any)

tcga <- avereps(tcga)

# Log2 transform

tcga <- log2(tcga + 1)

# Remove non-tumor samples (keep primary tumor; TCGA barcode 4th field starts with "0")

group <- sapply(strsplit(colnames(tcga), "\\-"), "[", 4)

group <- sapply(strsplit(group, ""), "[", 1)

group <- gsub("2", "1", group)

tcga <- tcga[, group == 0]

# Harmonize sample IDs to first 3 barcode fields (TCGA-XX-YYYY)

tcga <- t(tcga)

rownames(tcga) <- gsub("(.*?)\\-(.*?)\\-(.*?)\\-.*", "\\1\\-\\2\\-\\3", rownames(tcga))

tcga <- t(avereps(tcga))

## 2) Read and preprocess GEO

rt <- read.table(geoExpFile, header = TRUE, sep = "\t", check.names = FALSE)

rt <- as.matrix(rt)

rownames(rt) <- rt[, 1]

exp <- rt[, 2:ncol(rt)]

dimnames <- list(rownames(exp), colnames(exp))

geo <- matrix(as.numeric(as.matrix(exp)),

nrow = nrow(exp),

dimnames = dimnames)

# Collapse duplicated gene symbols (if any)

geo <- avereps(geo)

## 3) Keep common genes and apply ComBat

sameGene <- intersect(rownames(tcga), rownames(geo))

tcgaOut <- tcga[sameGene, ]

geoOut <- geo[sameGene, ]

# Combine and correct batch (1 = TCGA, 2 = GEO)

all <- cbind(tcgaOut, geoOut)

batchType <- c(rep(1, ncol(tcgaOut)), rep(2, ncol(geoOut)))

outTab <- ComBat(dat = all, batch = batchType, par.prior = TRUE)

# Split corrected matrices back

tcgaOut <- outTab[, colnames(tcgaOut)]

geoOut <- outTab[, colnames(geoOut)]

# Truncate negative values to zero

tcgaOut[tcgaOut < 0] <- 0

geoOut[geoOut < 0] <- 0

## 4) Export normalized expression matrices

tcgaTab <- rbind(ID = colnames(tcgaOut), tcgaOut)

write.table(tcgaTab, file = "TCGA.normalize.txt",

sep = "\t", quote = FALSE, col.names = FALSE)

geoTab <- rbind(ID = colnames(geoOut), geoOut)

write.table(geoTab, file = "GEO.normalize.txt",

sep = "\t", quote = FALSE, col.names = FALSE)

## 5) Export PRG-only matrices

gene <- read.table(geneFile, header = TRUE, sep = "\t", check.names = FALSE)

sameGenePRG <- intersect(as.vector(gene[, 1]), rownames(tcgaOut))

tcgaShareExp <- tcgaOut[sameGenePRG, ]

geoShareExp <- geoOut[sameGenePRG, ]

tcgaShareExp <- rbind(ID = colnames(tcgaShareExp), tcgaShareExp)

write.table(tcgaShareExp, file = "TCGA.share.txt",

sep = "\t", quote = FALSE, col.names = FALSE)

geoShareExp <- rbind(ID = colnames(geoShareExp), geoShareExp)

write.table(geoShareExp, file = "GEO.share.txt",

sep = "\t", quote = FALSE, col.names = FALSE)

**## Supplementary Code S4: ssGSEA scoring (Hallmark / Immune gene sets)**

library(limma)

library(GSVA)

library(GSEABase)

library(SummarizedExperiment)

expFile <- "TCGA.normalize.txt" # log2-normalized expression (genes x samples)

gmtFile <- "immune.gmt" # gene sets (Hallmark/Immune) in GMT format

rt <- read.table(expFile, header = TRUE, sep = "\t", check.names = FALSE)

rownames(rt) <- rt[,1]

exp <- as.matrix(rt[, -1])

exp <- avereps(exp)

geneSet <- getGmt(gmtFile, geneIdType = SymbolIdentifier())

ssgseaScore <- gsva(exp, geneSet, method = "ssgsea", kcdf = "Gaussian", abs.ranking = TRUE)

write.table(ssgseaScore, file = "ssGSEA_scores.txt", sep = "\t", quote = FALSE, col.names = NA)

**## Supplementary Code S5: GO + KEGG enrichment (clusterProfiler)**

library(clusterProfiler)

library(org.Hs.eg.db)

diffFile <- "riskDiff.txt" # first column = gene symbols

rt <- read.table(diffFile, header = TRUE, sep = "\t", check.names = FALSE)

genes <- unique(rt[,1])

entrez <- mget(genes, org.Hs.egSYMBOL2EG, ifnotfound = NA)

entrez <- as.character(entrez)

gene <- entrez[entrez != "NA"]

## KEGG enrichment

kegg <- enrichKEGG(gene = gene, organism = "hsa", pvalueCutoff = 0.05, qvalueCutoff = 0.05)

write.table(as.data.frame(kegg), file = "KEGG.txt", sep = "\t", quote = FALSE, row.names = FALSE)

## GO enrichment

go <- enrichGO(gene = gene, OrgDb = org.Hs.eg.db, ont = "ALL", pvalueCutoff = 0.05, qvalueCutoff = 0.05, readable = TRUE)

write.table(as.data.frame(go), file = "GO.txt", sep = "\t", quote = FALSE, row.names = FALSE)

**## Supplementary Code S6: MCP-counter analysis**

library(limma)

library(MCPcounter)

expFile <- "symbol.txt" # gene symbol expression matrix (genes x samples)

probeFile <- "probesets.txt"

geneFile <- "genesets.txt"

rt <- read.table(expFile, header = TRUE, sep = "\t", check.names = FALSE)

rownames(rt) <- rt[,1]

exp <- as.matrix(rt[, -1])

exp <- avereps(exp)

exp <- exp[rowMeans(exp) > 0, ]

## (Optional) keep primary tumor samples only (TCGA sample type = 01)

group <- sapply(strsplit(colnames(exp), "\\-"), "[", 4)

group <- sapply(strsplit(group, ""), "[", 1)

group <- gsub("2", "1", group)

exp <- exp[, group == "0"]

## harmonize sample IDs to patient barcodes

exp <- t(exp)

rownames(exp) <- gsub("(.*?)\\-(.*?)\\-(.*?)\\-.*", "\\1\\-\\2\\-\\3", rownames(exp))

exp <- t(avereps(exp))

## MCP-counter estimation

mcp <- MCPcounter.estimate(

exp,

featuresType = "HUGO_symbols",

probesets = read.table(probeFile, sep = "\t", stringsAsFactors = FALSE, colClasses = "character"),

genes = read.table(geneFile, sep = "\t", header = TRUE, stringsAsFactors = FALSE, colClasses = "character", check.names = FALSE)

)

out <- rbind(ID = colnames(mcp), mcp)

write.table(out, file = "MCPcounter.result.txt", sep = "\t", quote = FALSE, col.names = FALSE)
